# Efficacy and safety of early radiotherapy combined with first-line chemo-immunotherapy in extensive-stage small-cell lung cancer: a multi-center analysis

**DOI:** 10.3389/fimmu.2026.1738352

**Published:** 2026-01-22

**Authors:** Jingyi Jia, Ya Zeng, Yongling Ji, Hui Zhu, Rui Meng, Bing Xia, Yunfeng Wang, Tianle Shen, Xi Su, Tongfang Zhou, Yifei Lu, Lei Zhao, Zhangru Yang, Xiaolong Fu, Xuwei Cai

**Affiliations:** 1 Department of Radiation Oncology, Shanghai Chest Hospital, Shanghai Jiao Tong University School of Medicine, Shanghai, China; 2 Department of Radiation Oncology, Zhejiang Cancer Hospital, Hangzhou Institute of Medicine (HIM), Chinese Academy of Sciences, Hangzhou, China; 3 Department of Radiation Oncology, Shandong Cancer Hospital and Institute, Shandong First Medical University and Shandong Academy of Medical Science, Jinan, China; 4 Oncology Center, Union Hospital, Tongji Medical College, Huazhong University of Science and Technology, Wuhan, China; 5 Department of Thoracic Oncology, Hangzhou Cancer Hospital, Zhejiang University School of Medicine, and Cancer Center, Zhejiang University, Hangzhou, China

**Keywords:** efficacy, extensive-stage small-cell lung cancer, first-line chemo-immunotherapy, immunotherapy, radiotherapy, safety

## Abstract

**Backgrounds:**

This study aimed to evaluate efficacy and safety of early radiotherapy combined with first-line chemo-immunotherapy in patients with extensive-stage small-cell lung cancer (ES-SCLC).

**Methods:**

ES-SCLC patients treated with first-line chemo-immunotherapy from August 2018 to January 2024 at five centers were included. Patients receiving early radiotherapy administered before disease progression were categorized into RT group, and were further separately stratified by the receipt of thoracic radiotherapy (TRT) and extra thoracic radiotherapy (eTRT). Propensity score matching (PSM) was performed to balance potential bias.

**Results:**

Totally, 771 patients were enrolled. RT group exhibited significantly better progression-free survival (PFS, p<0.001) and overall survival (OS, p<0.001) compared to Non-RT group in the whole and post-PSM cohort. The survival benefit was also observed in TRT group versus Non-TRT group, and eTRT group versus Non-eTRT group. Exploratory analyses were conducted within the TRT group. Consolidative TRT was associated with superior PFS (p<0.001) and OS (p=0.006) compared to the concurrent treatment. Additionally, survival outcomes were comparable between patients receiving a biological effective dose (BED) below or above the median dose of 60Gy and the salvage dose of 39Gy. No significance was observed in overall adverse events between RT group and Non-RT group, despite a higher rate of all-grade pneumonitis in RT group (9.9% versus 4.2%, p=0.002).

**Conclusions:**

Early radiotherapy, either thoracic or extra thoracic radiotherapy, combined with first-line chemo-immunotherapy constituted an effective and well-tolerated strategy for patients with ES-SCLC. These findings warrant investigation in prospective randomized trials.

## Introduction

Small-cell lung cancer (SCLC), accounting for approximately 15% of all new lung cancer cases, is marked by its highly aggressive nature and poor prognosis (1). In the majority of cases, patients are initially diagnosed with metastatic extensive-stage disease (2). Etoposide-platinum chemotherapy has been the mainstay therapy for extensive-stage SCLC (ES-SCLC) over the past few decades, with an unfavorable median overall survival (OS) of approximately 10 months (3). Advent of immune checkpoint inhibitors (ICIs) has led to establishment of immunotherapy in conjunction with chemotherapy as the standard of care for ES-SCLC, as evidenced by landmark IMpower133, CASPIAN, CAPSTONG-1, ASTRUM-005, RATIONALE-312, as well as EXTENTORCH studies (4–9). This combination has been demonstrated to improve OS by 1–3 months compared to chemotherapy alone. Nevertheless, the enhancement was modest, and many patients would develop early resistance to systemic therapy within a few months (10). And a large proportion of patients with ES-SCLC would experience primary tumor and regional lymph nodes progression (11). Consequently, it’s eagerly anticipated for new regimens to address these issues.

Radiotherapy (RT) is a promising strategy for ES-SCLC. Before the emergence of ICIs, the phase III CREST study illuminated efficacy of radiotherapy in treatment of ES-SCLC. This study demonstrated that integration of thoracic radiotherapy with chemotherapy significantly improved 2-year OS rate (p=0.004) and induced an almost 50% reduction in intrathoracic recurrences compared to chemotherapy alone (12). In the era of immunotherapy, the value of radiotherapy has also been demonstrated with substantial evidence ranging from preclinical researches to real-world practices (13–16). Several retrospective studies have elucidated that addition of radiotherapy could improve survival outcomes of ES-SCLC patients receiving first-line chemo-immunotherapy (17–21). A recent prospective phase II study, which enrolled 67 ES-SCLC patients receiving first-line chemo-immunotherapy, has shown improvement of progression-free survival (PFS) and OS along with addition of consolidative thoracic radiotherapy (22). However, most current studies were presented with small sample sizes, and there is limited evidence on irradiated sites, optimal timing and dose of radiotherapy when combined with chemo-immunotherapy. This large, multi-center, real-world study aimed to evaluate the efficacy and safety of radiotherapy in ES-SCLC patients with first-line treatment of chemo-immunotherapy, with an attempt to identify optimal timing and dose of radiotherapy.

## Materials and methods

### Patients

In this cohort study, we reviewed medical records of patients who received first-line chemo-immunotherapy at five hospitals in China between August 2018 and January 2024. The inclusion criteria were: (I) aged 18 years or older, (II) cytologically or pathologically confirmed SCLC, (III) diagnosed with extensive stage according to Veterans Administration Lung Study Group [VALG] staging system, (IV) treated with chemo-immunotherapy as first-line therapy, and (V) Eastern Cooperative Oncology Group performance status (ECOG PS) of 0 or 1. The exclusion criteria were as follows: (I) a history of or current diagnosis of other malignancies, (II) received immunotherapy during limited-stage disease, and (III) lack of detailed treatment information. This study adhered to the principles of Declaration of Helsinki and was approved by the Ethical Review Boards and Institutional Review Boards of the participating facilities in this study. The informed consent process was aligned with standard clinical practice.

### Treatment

The eligible patients received first-line platinum-based double-agent chemotherapy regimens combined with either anti-PD1 or anti-PD-L1 immunotherapy. According to the receipt of early radiotherapy, patients were grouped into RT group and Non-RT group. Stratified by administration of thoracic radiotherapy (TRT) and extra thoracic radiotherapy (eTRT) before disease progression, patients were further separately categorized into: TRT group vs Non-TRT group, and eTRT group vs Non-eTRT group. The treatment regimens and adjustments were determined by physicians in accordance with guidelines of the National Comprehensive Cancer Network (NCCN) or the Chinese Society of Clinical Oncology (CSCO). Irradiated sites and doses were determined by physicians based on comprehensive evaluation of each patient’s clinical data. Given the heterogeneous radiation fractionations, the biological effective dose (BED) formula was used: BED = nd × [1 + d/(α/β)] (23), where n represents the number of fractions, d is the dose per fraction, and α/β is set to 10. Moreover, according to the prescribed dose per fraction, radiotherapy was classified into conventional fractionated RT (1.8-2.2 Gy per fraction), hyperfractionated RT (≤1.5 Gy per fraction) and hypofractionated RT (≥2.5 Gy per fraction) (24). In order to further investigate the optimal timing of thoracic radiotherapy, the TRT group was subdivided into concurrent TRT subset (≤4 cycles of systemic therapy) and consolidative TRT subset (>4 cycles of systemic therapy) based on the interval from the initiation of chemo-immunotherapy to thoracic radiotherapy. For exploratory analysis of radiation dose, the TRT group was categorized into BED<60Gy group and BED≥60Gy group separated by median BED dose 60Gy, or BED ≤ 39Gy group and BED>39Gy group based on salvage prescription.

### Assessment

PFS was defined as the interval from the date of initiation of immunotherapy to disease progression, death or the last-known follow-up date. OS was defined as the interval from the day of initiation of immunotherapy to the date of death due to any cause or the last follow-up date. The treatment-related adverse events (TRAEs) were evaluated according to the National Cancer Institute’s Common Terminology Criteria for Adverse Events (NCI-CTCAE, version 5.0).

### Statistical analysis

Continuous variables were presented as median with interquartile range (IQR). Categorical variables were presented as frequency and percentage. The Student’s t test or Chi-square test was used for comparison as appropriate. PFS and OS were estimated using the Kaplan-Meier method and compared via the log-rank test. Univariate and multivariate analyses were performed using Cox proportional-hazards models. Subgroup analyses were conducted. Propensity score matching (PSM) was performed using baseline characteristics with p-value less than 0.05 to minimize bias, with a caliper of 0.02 between groups. All statistical analyses were conducted using R Studio (version 4.4.1, R Core Team, Austria), with two-sided p-values and a significance level of 0.05.

## Results

### Patients

From August 2018 to January 2024, a total of 771 patients with ES-SCLC receiving first-line chemo-immunotherapy were enrolled in this study. Among them, 292 patients received radiotherapy before disease progression and were categorized into RT group, and the other 479 patients in Non-RT group. The baseline characteristics of patients were listed in **Table 1**. A total of 343 (44.5%) patients aged more than 65 years and 670 (86.9%) patients were male. At baseline, 130 patients (16.9%) had brain metastases, 111 patients (14.4%) had lung metastases, 219 patients (28.4%) had bone metastases and 215 patients (27.9%) had liver metastases. 507 patients (65.8%) received anti-PDL1 immunotherapy, while the others received anti-PD1 treatment.

**Table 1:** Baseline characteristics of RT group and Non-RT group before and after PSM.

Characteristics	Total	Before PSM			After PSM		
		RT(N = 292)	Non-RT(N = 479)	p	RT(N = 247)	Non-RT(N = 247)	p
Age, n(%)							
<65 years	428 (55.5)	179 (61.3)	249 (52.0)	0.014	151 (61.1)	149 (60.3)	0.927
≥65 years	343 (44.5)	113 (38.7)	230 (48.0)		96 (38.9)	98 (39.7)	
Gender, n(%)							
female	101 (13.1)	41 (14.0)	60 (12.5)	0.621	30 (12.1)	33 (13.4)	0.787
male	670 (86.9)	251 (86.0)	419 (87.5)		217 (87.9)	214 (86.6)	
Smoker, n(%)							
no	288 (37.4)	106 (36.3)	182 (38.0)	0.693	87 (35.2)	96 (38.9)	0.456
yes	483 (62.6)	186 (63.7)	297 (62.0)		160 (64.8)	151 (61.1)	
Brain metastasis, n(%)							
no	641 (83.1)	203 (69.5)	438 (91.4)	<0.001	203 (82.2)	206 (83.4)	0.812
yes	130 (16.9)	89 (30.5)	41 (8.6)		44 (17.8)	41 (16.6)	
Lung metastasis, n(%)							
no	660 (85.6)	267 (91.4)	393 (82.0)	<0.001	224 (90.7)	229 (92.7)	0.514
yes	111 (14.4)	25 (8.6)	86 (18.0)		23 (9.3)	18 (7.3)	
Bone metastasis, n(%)							
no	552 (71.6)	225 (77.1)	327 (68.3)	0.011	184 (74.5)	183 (74.1)	1.000
yes	219 (28.4)	67 (22.9)	152 (31.7)		63 (25.5)	64 (25.9)	
Liver metastasis, n(%)							
no	556 (72.1)	235 (80.5)	321 (67.0)	<0.001	192 (77.7)	189 (76.5)	0.830
yes	215 (27.9)	57 (19.5)	158 (33.0)		55 (22.3)	58 (23.5)	
Type of ICIs, n(%)							
anti-PDL1	507 (65.8)	199 (68.2)	308 (64.3)	0.310	173 (70.0)	168 (68.0)	0.697
anti-PD1	264 (34.2)	93 (31.8)	171 (35.7)		74 (30.0)	79 (32.0)	

RT, radiotherapy; ICIs, immune checkpoint inhibitors; PDL1, programmed cell death ligand 1; PD1, programmed cell death 1; PSM, propensity score matching.

To be more specific, 212 patients received early thoracic radiotherapy before disease progression in the TRT group, and the other 559 patients in the Non-TRT group. 203 patients had a documented TRT regimen. Among them, 97 patients (47.8%) received conventional fractionated TRT (40.0-61.6 Gy at 1.8-2.2 Gy per fraction, BED = 48-75.15 Gy), 33 patients (16.2%) received hyperfractionated RT (39.0-60.0Gy at 1.5Gy per fraction, BED = 44.85-68.0Gy) and 73 patients (36.0%) received hypofractionated TRT (15.0-84.0Gy at 2.5-15Gy per fraction, BED = 19.5-187.5Gy). The eTRT group consisted of 90 patients who underwent extra thoracic radiotherapy before disease progression. The predominantly irradiated metastatic site was brain (n=65), followed by bone (n=17). Totally, 12 patients received radiotherapy at more than one site.

### Survival outcomes

**Figure 1:**
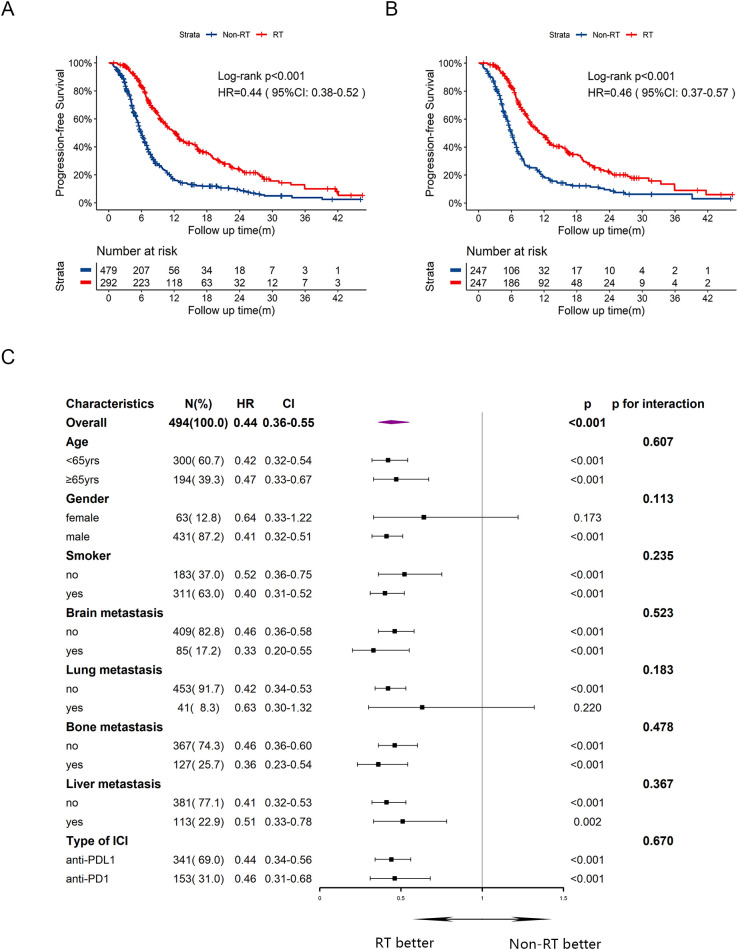
Kaplan-Meier survival curves of PFS in RT group and Non-RT group before and after PSM, and subgroup analysis of PFS in the post-PSM cohort. **(A)** Kaplan-Meier curve of PFS before PSM. **(B)** Kaplan-Meier curve of PFS after PSM. **(C)** Subgroup analysis of PFS after PSM. yrs, years; m, months; PFS, progression-free survival; HR, hazard ratio; CI, confidence interval; RT, radiotherapy.

At the data cutoff, the median follow-up period was 18.5 (IQR: 17.4-20.4) months. The median PFS was significantly longer in RT group compared to Non-RT group (12.1 months vs 6.0 months), with a hazard ratio (HR) of 0.44 (**Figure 1A**, 95% CI:0.38-0.52, p<0.001). The one-year PFS rate was 50.4% in RT group versus 16.6% in Non-RT group, while the two-year PFS rate was 23.8% versus 9.2%, respectively. After PSM, improved PFS was also observed, with median PFS of 11.0 months in RT group and 6.0 months in Non-RT group (**Figure 1B**, HR = 0.46, 95%CI:0.37-0.57, p<0.001). Univariate and multivariate cox analysis (**Supplementary Table 1**) for PFS revealed that male sex (HR = 1.59, 95%CI:1.22-2.08, p<0.001), and baseline brain (HR = 1.32, 95%CI:1.04-1.67, p=0.024), bone (HR = 1.40, 95%CI:1.16-1.68, p<0.001) or liver metastasis (HR = 1.25, 95%CI:1.04-1.50, p=0.018) were associated with poorer PFS, while early radiotherapy (HR = 0.41, 95%CI:0.34-0.50, p<0.001) was correlated with improved PFS. Subgroup analysis for PFS (**Figure 1C**) indicated that most subgroups could benefit from early radiotherapy.

**Figure 2:**
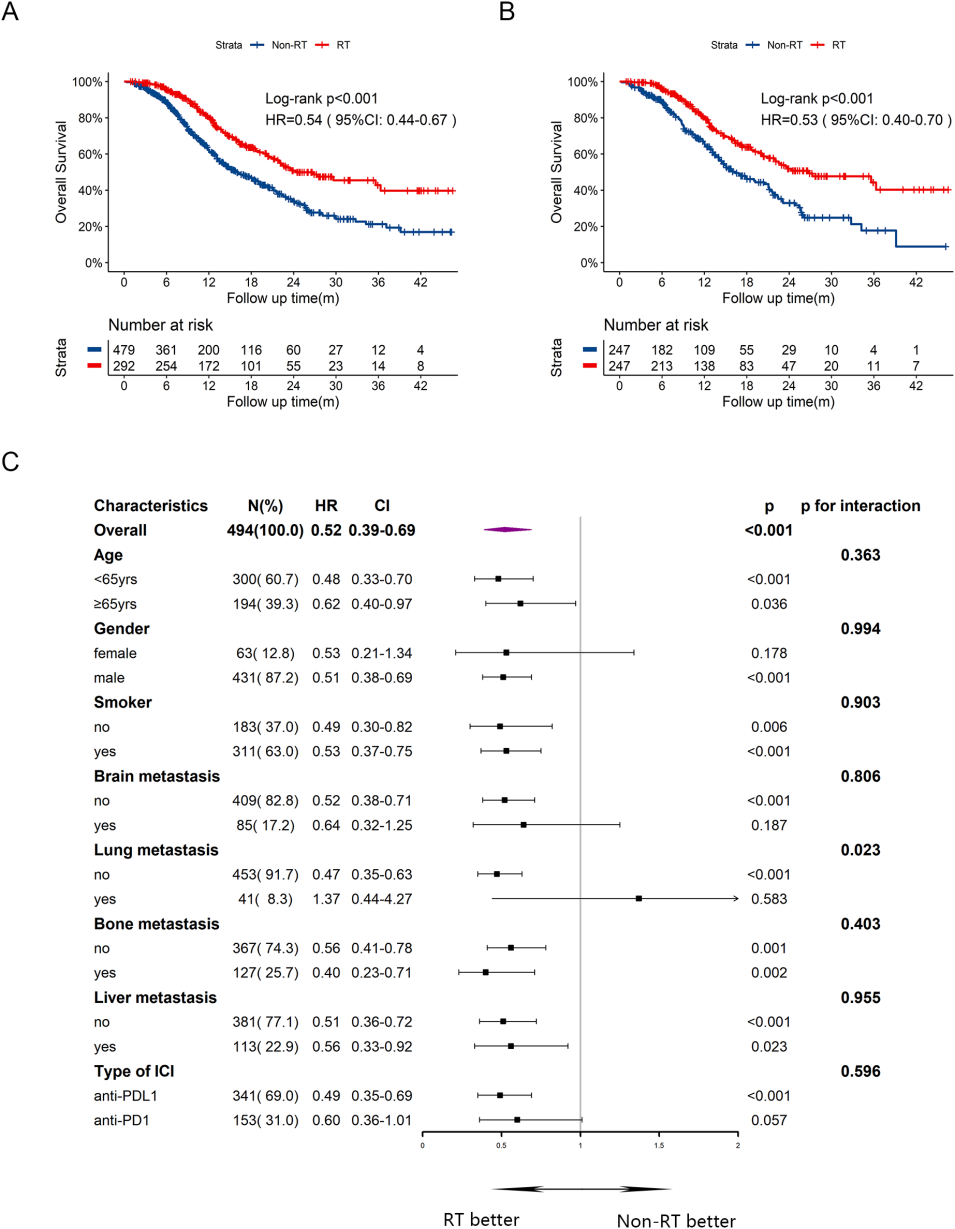
Kaplan-Meier survival curves of OS in RT group and Non-RT group before and after PSM, and subgroup analysis of OS in the post-PSM cohort. **(A)** Kaplan-Meier curve of OS before PSM. **(B)** Kaplan-Meier curve of OS after PSM. **(C)** Subgroup analysis of OS after PSM. yrs, years; m, months; OS, overall survival; HR, hazard ratio; CI, confidence interval; RT, radiotherapy.

In terms of OS, the median OS was 24.3 months in RT group and 16.0 months in Non-RT group, with a HR of 0.54 (**Figure 2A**, 95%CI:0.44-0.67, p<0.001). The one-year and two-year OS rates were 80.4% and 50.9%, respectively, in RT group, compared to 62.2% and 34.1%, respectively, in Non-RT group. After PSM, patients in RT group still had prolonged OS with median time of 26.8 months, compared to 16.3 months in Non-RT group (**Figure 2B**, HR = 0.53, 95%CI:0.40-0.70, p<0.001). In multivariate cox analysis (**Supplementary Table 1**) for OS, male sex (HR = 1.82, 95%CI:1.25-2.64, p=0.002) and liver metastasis at baseline (HR = 1.64, 95%CI:1.30-2.05, p<0.001) were independently risk factors for OS. Early radiotherapy was confirmed as an independently favorable predictor for OS (HR = 0.57, 95%CI: 0.45-0.72, p<0.001). Subgroup analysis for OS (**Figure 2C**) exhibited that early radiotherapy improved OS in most subgroups except for female and those with brain or lung metastasis at baseline, or receiving anti-PDL1 therapy. Notably, patients with bone or liver metastasis at baseline could also benefit from early radiotherapy.

**Figure 3:**
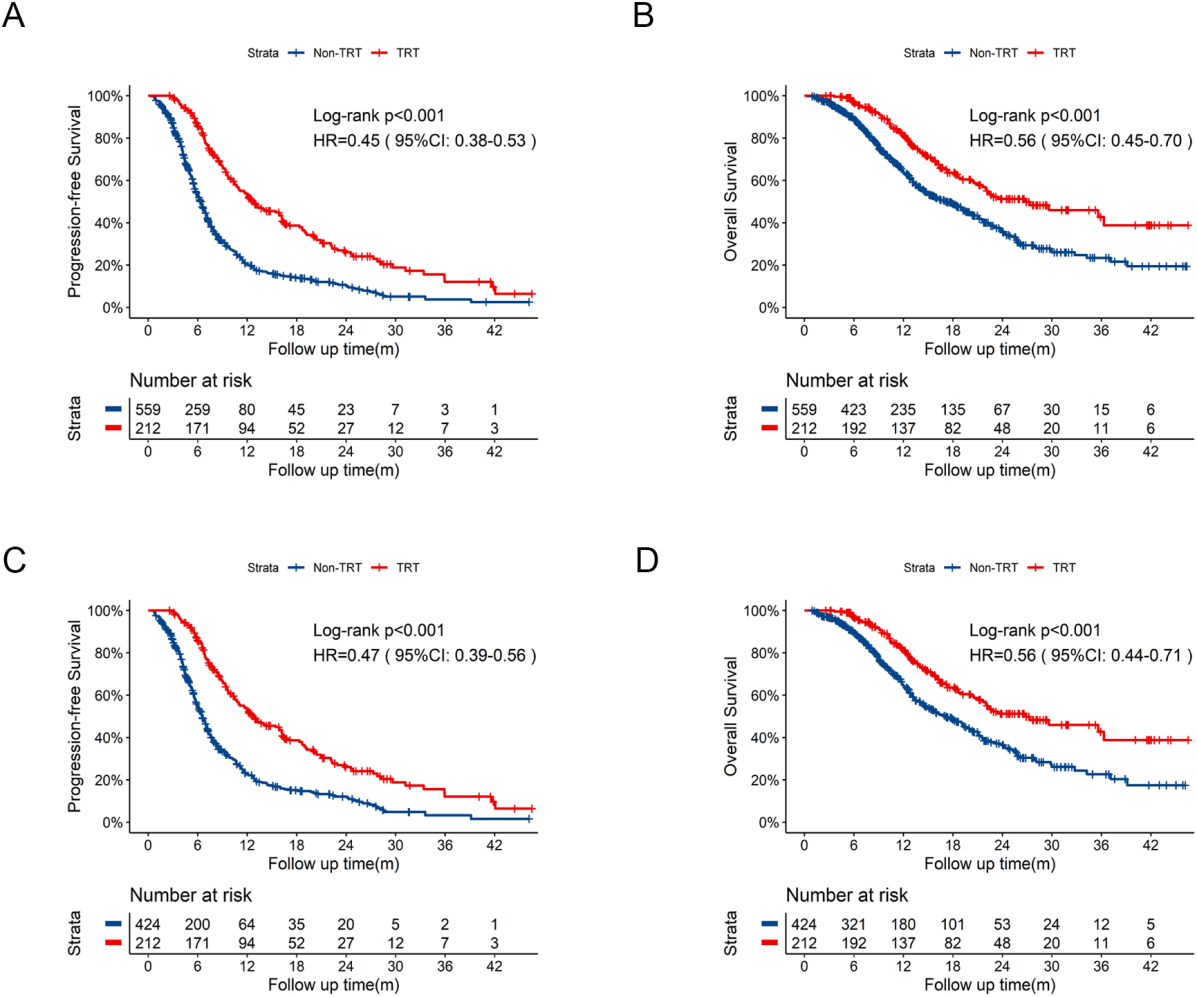
Kaplan-Meier survival curves of PFS and OS in TRT group compared to Non-TRT group before and after PSM. **(A)** Kaplan-Meier curve of PFS in TRT group compared to Non-TRT group before PSM. **(B)** Kaplan-Meier curve of OS in TRT group compared to Non-TRT group before PSM. **(C)** Kaplan-Meier curve of PFS in TRT group compared to Non-TRT group after PSM. **(D)** Kaplan-Meier curve of OS in TRT group compared to Non-TRT group after PSM. PSM, propensity score matching; PFS, progression-free survival; OS, overall survival; HR, hazard ratio; CI, confidence interval; TRT, thoracic radiotherapy; m, months.

With the aim to define the role of thoracic radiotherapy, comparisons between TRT group (N = 212) and Non-TRT group (N = 559) were performed (**Supplementary Table 2**). Similarly, TRT group exhibited significantly longer PFS (**Figure 3A**, 12.7 months vs 6.5 months, HR = 0.45, 95% CI:0.38-0.53, p<0.001) and OS (**Figure 3B**, 26.8 months vs 17.3 months, HR = 0.56, 95%CI:0.45-0.70, p<0.001) compared with those in the Non-TRT group. After PSM, significant improvements in PFS (**Figure 3C**, 12.7 months vs 6.5 months, HR = 0.47, 95%CI:0.39-0.56, p<0.001) and OS (**Figure 3D**, 26.8 months vs 17.1 months, HR = 0.56, 95%CI:0.44-0.71, p<0.001) persisted in TRT group.

**Figure 4:**
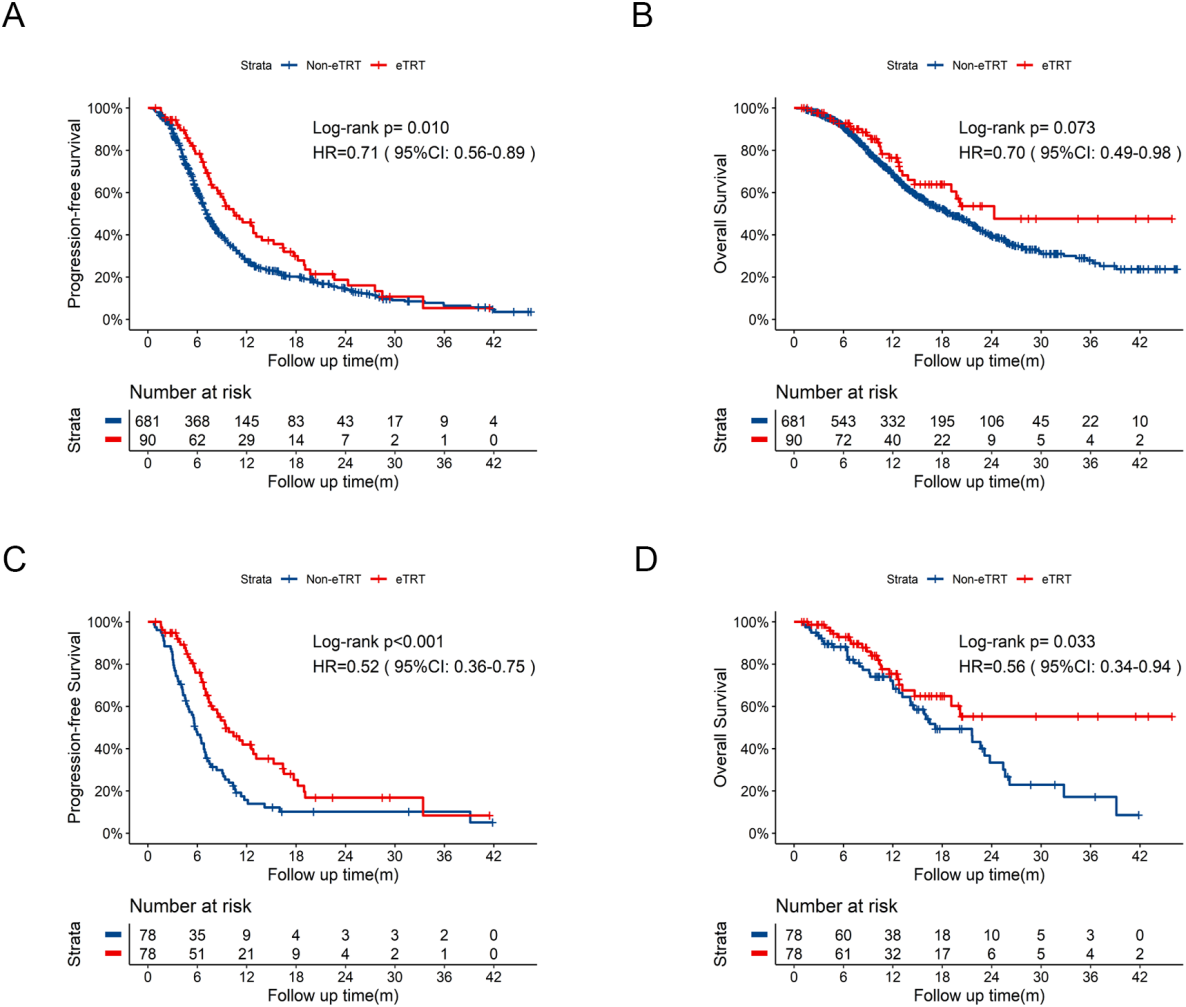
Kaplan-Meier survival curves of PFS and OS in eTRT group compared to Non-eTRT group before and after PSM. **(A)** Kaplan-Meier curve of PFS in eTRT group compared to Non-eTRT group before PSM. **(B)** Kaplan-Meier curve of OS in eTRT group compared to Non-eTRT group before PSM. **(C)** Kaplan-Meier curve of PFS in eTRT group compared to Non-eTRT group after PSM. **(D)** Kaplan-Meier curve of OS in eTRT group compared to Non-eTRT group after PSM. PSM, propensity score matching; PFS, progression-free survival; OS, overall survival; HR, hazard ratio; CI, confidence interval; eTRT, extra thoracic radiotherapy; m, months.

Further comparisons between eTRT group (N = 90) and Non-eTRT group (N = 681) were conducted. eTRT group exhibited an extended PFS of 10.5 months vs 7.2 months (**Figure 4A**, HR = 0.71, 95%CI: 0.56-0.89, p=0.010) compared to Non-eTRT group, whereas OS was not statistically significant of 24.3 months vs 19.0 months (**Figure 4B**, HR = 0.70, 95%CI:0.49-0.98, p=0.073). With well-balanced baseline characteristics in **Supplementary Table 3**, significantly improved PFS (**Figure 4C**, HR = 0.52, 95%CI: 0.36-0.75, p<0.001) and OS (**Figure 4D**, HR = 0.56, 95%CI: 0.34-0.94, p=0.033) both were observed in eTRT group.

### Exploratory analysis

To further explore optimal timing and dose of thoracic radiotherapy, we conducted additional analysis in subgroups of TRT group. TRT group was divided into two subsets based on the interval from the initiation of immunotherapy to radiotherapy: concurrent TRT subset (≤4 cycles of systemic therapy) and consolidative TRT subset (>4 cycles of systemic therapy). Also, PSM was performed and the baseline characteristics were listed in **Supplementary Table 4**. In the PSM cohort, consolidative TRT showed significantly prolonged median PFS (**Supplementary Figure 1A**) of 18.3 months vs 8.8 months (HR = 0.50, 95%CI:0.34-0.72, p<0.001). Consolidative TRT was associated with significantly longer OS (**Supplementary Figure 1B**, 35.6 vs 20.9 months; HR = 0.52, 95%CI:0.32-0.84, p=0.006) compared to the concurrent TRT subset.

A total of 203 patients in the TRT group with recorded BED data were included to assess the correlation between BED and survival outcomes. The median BED of TRT group was 60Gy. With comparable characteristics of those who received BED≥60Gy and BED<60Gy (**Supplementary Table 5**), no significant benefit was observed in PFS (**Supplementary Figure 1C**, p=0.832) and OS (**Supplementary Figure 1D**, p=0.210) associated with higher BED. Additionally, subsets with BED ≤ 39Gy or BED>39Gy in TRT group were compared, of which baseline characteristics were shown in **Supplementary Table 6**. After PSM, no significant difference was observed in PFS (**Supplementary Figure 1E**, p=0.413) and OS (**Supplementary Figure 1F**, p=0.447).

### Safety

At the cut-off date, no treatment-related deaths were recorded. The toxicity profiles were shown in **Table 2**. Patients in Non-RT group experienced more treated-related adverse events compared to RT group (p<0.001). Grade 3 or higher adverse events between two groups were similar (p=0.198). The most common treatment-related adverse events were hematological toxicities, with 60 patients (20.5%) in RT group and 190 patients (39.7%) in Non-RT group. Additionally, pneumonitis occurred in 29 patients (9.9%) in RT group and 20 patients (4.2%) in Non-RT group, with a statistically significance (p=0.002). While the incidence of grade 3 or higher pneumonitis was not significant between the two groups (5.5% vs 2.5%, p=0.052).

**Table 2:** Treatment-related adverse events in RT and Non-RT group.

Adverse events	RT (N = 292,%)	Non-RT (N = 479,%)	p
All grades			
Adverse events	94 (32.2)	233 (48.6)	<0.001
Hematological toxicities	60 (20.5)	190 (39.7)	<0.001
Pneumonitis	29 (9.9)	20 (4.2)	0.002
Hepatic dysfunction	6 (2.1)	15 (3.1)	0.507
Rash	5 (1.7)	17 (3.5)	0.207
Esophagitis	4 (1.4)	2 (0.4)	0.300
Hypothyroidism	5 (1.7)	11 (2.3)	0.771
Diarrhea	4 (1.4)	1 (0.2)	0.137
Nausea or vomiting	4 (1.4)	12 (2.5)	0.417
Fatigue	4 (1.4)	9 (1.9)	0.807
Dizziness	1 (0.3)	4 (0.8)	0.716
Hyperthyroidism	1 (0.3)	1 (0.2)	1.000
Constipation	1 (0.3)	4 (0.8)	0.716
≥Grade 3			
Adverse events≥Grade 3	48 (16.4)	98 (20.5)	0.198
Hematological toxicities	33 (11.3)	86 (18.0)	0.017
Pneumonitis	16 (5.5)	12 (2.5)	0.052
Hepatic dysfunction	2 (0.7)	1 (0.2)	0.664
Rash	2 (0.7)	2 (0.4)	1.000
Diarrhea	1 (0.3)	1 (0.2)	1.000

RT, radiotherapy.

## Discussion

To our knowledge, this multi-center study is the largest real-world study to explore the efficacy and safety of early radiotherapy combined with first-line chemo-immunotherapy in ES-SCLC. The results demonstrated that early radiotherapy, whether thoracic or extra thoracic, conferred a superior survival advantage in patients with ES-SCLC receiving first-line chemo-immunotherapy with a tolerable safety profile.

ES-SCLC is an aggressive malignancy with poor prognosis. In the era of chemotherapy, Han et al. proposed that radiotherapy was essential for local disease control, especially administered within 6 cycles of chemotherapy (25). On the other hand, the phase III CREST trial demonstrated the efficacy of consolidative radiotherapy following chemotherapy, with great improvement in PFS, OS and intrathoracic disease control (12). With the emergence of immune checkpoint inhibitors (ICIs), chemo-immunotherapy has been established as the standard of care for ES-SCLC (4–8). However, the role of radiotherapy has not been well defined under the context of chemo-immunotherapy, mainly due to lack of permission in most phase III trials with concerns on potential increased risk of adverse events. Numerous preclinical researches have elucidated that radiotherapy could induce tumor-specific antigen exposure (26), reshape tumor microenvironment (27) and trigger systemic anti-tumor immune response (14, 17), laying the foundation for combination of radiotherapy and immunotherapy. In clinical settings, a recent meta-analysis, which encompassed 12 retrospective studies and 3 prospective studies, highlighted the efficacy of consolidative thoracic radiotherapy combined with first-line chemo-immunotherapy (28). Notably, in this large real-world study, our findings specifically emphasized that radiotherapy should be regarded as an early-intervention strategy, and reinforced the essential role of consolidative thoracic radiotherapy in treatment for ES-SCLC.

The optimal prescription of thoracic radiotherapy are still in suspense. Although a palliative dose of 30Gy in 10 fractions was suggested in the CREST trial, this was accompanied by an unsatisfactory intrathoracic recurrence rate of 41.7%, revealing the potential for dose escalation to improve local control (12). Under the context of chemo-immunotherapy, this real-word study showed lack of survival benefit from dose escalation (beyond BED of 60Gy or 39Gy), revealing the need for individualized dose selection. The phase II MATCH trial explored the feasibility of low-dose thoracic radiotherapy at a dosage of 15Gy in 5 fractions plus chemo-immunotherapy for patients with ES-SCLC (29). The ongoing phase III RAPTOR (NRG-LU007) study aimed to investigate different radiation dose adopted for different sites, specifically with definitive dose targeted at thoracic disease and palliative dose at metastatic lesions. The results are eagerly awaited (30).

Existing researches have emphasized the pivotal role of thoracic radiotherapy in ES-SCLC, while irradiation towards metastatic lesions is commonly considered as a symptomatic treatment. The phase II PEMBRO-RT study was conducted in patients with metastatic non-small-cell lung cancer. In this trial, those who received SBRT on a single, primarily metastatic site, followed by immunotherapy, had a doubling of the overall response rate, indicating the viability of early intervention of radiotherapy to metastatic sites (31). In our study, patients with ES-SCLC stratified by extra thoracic radiotherapy were directly compared. Align with our previous findings on thoracic radiotherapy, early extra thoracic radiotherapy exhibited improved survival outcomes. In summary, early radiotherapy, either thoracic or extra thoracic, combined with chemo-immunotherapy may be an ideal first-line treatment paradigm for ES-SCLC.

Generally, the safety profile is acceptable. In this study, the whole treated-related AEs were not significantly increased in patients receiving early radiotherapy, albeit with higher incidence of pneumonitis. However, there’s no significance in grade≥3 pneumonitis. Consistently, previous studies also exhibited manageable and primarily low-grade adverse events. Radiotherapy was reported with elevated risk of all-grade pneumonitis with incidence of about 13.8%-25.0%, and the grade 3 or higher pneumonitis was ranging from 2.6% to 6.5% (20–22, 32, 33). In summary, first-line radiotherapy combined with chemo-immunotherapy appears to be safe. Owing to limited evidence from prospective study, the safety of the combined regimen requires careful consideration.

This study has several limitations. First, although it was based on a large multi-center cohort, the non-randomized design may have introduced selection bias and unmeasured confounding factors. Second, heterogeneity existed in radiotherapy techniques, target volumes, and dosing regimens across centers, which might have influenced survival and toxicity outcomes. Additionally, the analysis was limited by missing clinical data, such as the application of prophylactic cranial irradiation, cycles of systemic therapy, and later-line treatments, which might have influenced the survival analyses. Finally, the limited follow-up duration precluded a definitive assessment of long-term survival and late toxicities. Due to the defects, the findings should be interpreted with caution.

In conclusion, early radiotherapy, either thoracic or extra thoracic radiotherapy, combined with first-line chemo-immunotherapy constituted an effective and well-tolerated strategy for patients with ES-SCLC. These findings warrant further investigation in large-scale prospective randomized trials in the future.

## Data Availability

The raw data supporting the conclusions of this article will be made available by the authors, without undue reservation.
