# Correction: Efficacy and safety of early radiotherapy combined with first-line chemo-immunotherapy in extensive-stage small-cell lung cancer: a multi-center analysis

**DOI:** 10.3389/fimmu.2026.1818306

**Published:** 2026-03-10

**Authors:** Jingyi Jia, Ya Zeng, Yongling Ji, Hui Zhu, Rui Meng, Bing Xia, Yunfeng Wang, Tianle Shen, Xi Su, Tongfang Zhou, Yifei Lu, Lei Zhao, Zhangru Yang, Xiaolong Fu, Xuwei Cai

**Affiliations:** 1 Department of Radiation Oncology, Shanghai Chest Hospital, Shanghai Jiao Tong University School of Medicine, Shanghai, China; 2 Department of Radiation Oncology, Zhejiang Cancer Hospital, Hangzhou Institute of Medicine (HIM), Chinese Academy of Sciences, Hangzhou, China; 3 Department of Radiation Oncology, Shandong Cancer Hospital and Institute, Shandong First Medical University and Shandong Academy of Medical Science, Jinan, China; 4 Oncology Center, Union Hospital, Tongji Medical College, Huazhong University of Science and Technology, Wuhan, China; 5 Department of Thoracic Oncology, Hangzhou Cancer Hospital, Zhejiang University School of Medicine, and Cancer Center, Zhejiang University, Hangzhou, China

**Keywords:** efficacy, extensive-stage small-cell lung cancer, first-line chemo-immunotherapy, immunotherapy, radiotherapy, safety

Incorrect PFS rates numerical values were reported.

A correction has been made to the section “*Results, Survival outcomes,* Paragraph 1”:

“The one-year PFS rate was 50.4% in RT group versus 16.6% in Non-RT group, while the two-year PFS rate was 23.8% versus 9.2%, respectively.”

Incorrect OS rates numerical values were reported.

A correction has been made to the section “*Results, Survival outcomes,* Paragraph 2”:

“The one-year and two-year OS rates were 80.4% and 50.9%, respectively, in RT group, compared to 62.2% and 34.1%, respectively, in Non-RT group.”

The PFS data after PSM was incorrectly copied as OS data.

A correction has been made to the section “*Results, Survival outcomes,* Paragraph 3”:

“After PSM, significant improvements in PFS (**Figures 3C**, 12.7 months vs 6.5 months, HR = 0.47, 95%CI:0.39-0.56, p<0.001)”

The original version of this article has been updated.

