# Supplementary material for: Efficacy and safety of early radiotherapy combined with first-line chemo-immunotherapy in extensive-stage small-cell lung cancer: a multi-center analysis
Source: Front Immunol. 2026 Jan 22;17:1738352. doi: 10.3389/fimmu.2026.1738352 (PMC12872522; doi:10.3389/fimmu.2026.1738352)
Supplement: Supplementary file 1 [file DataSheet1.pdf]

Supplementary Material

Supplementary Figures and Tables

Supplementary Table 1. Univariate and multivariate cox analysis of PFS and OS.

Characteristics	PFS				OS			
	Univariate Cox		Multivariate Cox		Univariate Cox		Multivariate Cox	
	HR(95%CI)	p	HR(95%CI)	p	HR(95%CI)	p	HR(95%CI)	p
RT v.s Non-RT	0.43(0.36-0.51)	<0.001	0.41(0.34 - 0.50)	p<0.001	0.54(0.43-0.68)	p<0.001	0.57(0.45-0.72)	p<0.001
Age, years, (≥65 v.s <65)	1.05(0.89-1.23)	0.591	NA	NA	1.18(0.95-1.47)	0.128	NA	NA
Gender(male v.s female)	1.50(1.15-1.95)	0.002	1.59(1.22 - 2.08)	0.001	1.85(1.27-2.67)	0.001	1.82(1.25-2.64)	0.002
Smoker (yes v.s no)	1.03(0.87-1.22)	0.760	NA	NA	1.19(0.94-1.49)	0.141	NA	NA
Brain metastasis (yes v.s no)	0.80(0.64-0.99)	0.043	1.32(1.04-1.67)	0.024	0.88(0.66-1.17)	0.383	NA	NA
Lung metastasis (yes v.s no)	1.35(1.07-1.70)	0.010	1.06(0.84-1.34)	0.635	1.13(0.83-1.53)	0.445	NA	NA
Bone metastasis (yes v.s no)	1.46(1.23-1.75)	<0.001	1.40(1.16 - 1.68)	p<0.001	1.33(1.05-1.67)	0.016	1.18(0.94-1.49)	0.151
Liver metastasis (yes v.s no)	1.40(1.17-1.67)	<0.001	1.25(1.04 - 1.50)	0.018	1.81(1.45-2.27)	p<0.001	1.64(1.30-2.05)	p<0.001
anti-PD1 v.s anti-PDL1	1.12(0.94-1.33)	0.188	NA	NA	1.06(0.85-1.34)	0.597	NA	NA

Abbreviation: RT, radiotherapy; PDL1, programmed cell death 1 ligand 1; PD1, programmed cell death 1; HR, hazard ratio; CI, confidence interval; NA, not available.

Supplementary Table 2. Baseline characteristics of TRT group and Non-TRT group before and after PSM.

Characteristics	Total	Before PSM			After PSM		
		TRT (N=212)	Non-TRT (N=559)	p	TRT (N=212)	Non-TRT (N=424)	p
**Age, n(%)**							
<65 years	428(55.5)	133 (62.7)	295 (52.8)	0.016	133 (62.7)	239 (56.4)	0.147
≥65 years	343(44.5)	79 (37.3)	264 (47.2)		79 (37.3)	185 (43.6)	
**Gender, n(%)**							
female	101(13.1)	27 (12.7)	74 (13.2)	0.948	27 (12.7)	58 (13.7)	0.837
male	670(86.9)	185 (87.3)	485 (86.8)		185 (87.3)	366 (86.3)	
**Smoker, n(%)**							
no	288(37.4)	66 (31.1)	222 (39.7)	0.034	66 (31.1)	158 (37.3)	0.150
yes	483(62.6)	146 (68.9)	337 (60.3)		146 (68.9)	266 (62.7)	
**Brain metastasis, n(%)**							
no	641(83.1)	176 (83.0)	465 (83.2)	1.000	176 (83.0)	342 (80.7)	0.540
yes	130(16.9)	36 (17.0)	94 (16.8)		36 (17.0)	82 (19.3)	
**Lung metastasis, n(%)**							
no	660(85.6)	200 (94.3)	460 (82.3)	<0.001	200 (94.3)	400 (94.3)	1.000
yes	111(14.4)	12 (5.7)	99 (17.7)		12 (5.7)	24 (5.7)	
**Bone metastasis, n(%)**							
no	552(71.6)	168 (79.2)	384 (68.7)	0.005	168 (79.2)	320 (75.5)	0.336
yes	219(28.4)	44 (20.8)	175 (31.3)		44 (20.8)	104 (24.5)	
**Liver metastasis, n(%)**							
no	556(72.1)	168 (79.2)	388 (69.4)	0.009	168 (79.2)	320 (75.5)	0.336
yes	215(27.9)	44 (20.8)	171 (30.6)		44 (20.8)	104 (24.5)	

Abbreviation: TRT, thoracic radiotherapy; ICIs, immune checkpoint inhibitors; PDL1, programmed cell death 1 ligand 1; PD1, programmed cell death 1; PSM, propensity score matching.

Supplementary Table 3. Baseline characteristics of eTRT group and Non-eTRT group before and after PSM.

Characteristics	Before PSM			After PSM		
	eTRT (N=90)	Non-eTRT (N=681)	p	eTRT (N=78)	Non-eTRT (N=78)	p
**Age, n(%)**						
<65 years	52 (57.8)	376 (55.2)	0.728	48 (61.5)	44 (56.4)	0.625
≥65 years	38 (42.2)	305 (44.8)		30 (38.5)	34 (43.6)	
**Gender, n(%)**						
female	17 (18.9)	84 (12.3)	0.117	13 (16.7)	14 (17.9)	1.000
male	73 (81.1)	597 (87.7)		65 (83.3)	64 (82.1)	
**Smoker, n(%)**						
no	45 (50.0)	243 (35.7)	0.012	37 (47.4)	33 (42.3)	0.629
yes	45 (50.0)	438 (64.3)		41 (52.6)	45 (57.7)	
**Brain metastasis, n(%)**						
no	30 (33.3)	611 (89.7)	<0.001	30 (38.5)	30 (38.5)	1.000
yes	60 (66.7)	70 (10.3)		48 (61.5)	48 (61.5)	
**Lung metastasis, n(%)**						
no	76 (84.4)	584 (85.8)	0.862	64 (82.1)	69 (88.5)	0.366
yes	14 (15.6)	97 (14.2)		14 (17.9)	9 (11.5)	
**Bone metastasis, n(%)**						
no	65 (72.2)	487 (71.5)	0.987	56 (71.8)	56 (71.8)	1.000
yes	25 (27.8)	194 (28.5)		22 (28.2)	22 (28.2)	
**Liver metastasis, n(%)**						
no	75 (83.3)	481 (70.6)	0.016	66 (84.6)	58 (74.4)	0.165
yes	15 (16.7)	200 (29.4)		12 (15.4)	20 (25.6)	
**TRT, n(%)**						
TRT	80 (88.9)	479 (70.3)	<0.001	68 (87.2)	68 (87.2)	1.000
Non-TRT	10 (11.1)	202 (29.7)		10 (12.8)	10 (12.8)	

Abbreviation: PSM, propensity score matching; IQR, interquartile range; BED, biological effective dose; eTRT, extrathoracic radiotherapy.

Supplementary Table 4. Baseline characteristics of subsets with consolidative TRT (>4 cycles) and concurrent TRT (≤4 cycles) in TRT group before and after PSM.

Characteristics	Before PSM			After PSM		
	>4 cycles(N=124)	≤4 cycles(N=88)	p	>4 cycles(N=88)	≤4 cycles(N=88)	p
**Age, n(%)**						
<65 years	79 (63.7)	54 (61.4)	0.838	59 (67.0)	54 (61.4)	0.529
≥65 years	45 (36.3)	34 (38.6)		29 (33.0)	34 (38.6)	
**Gender, n(%)**						
female	13 (10.5)	14 (15.9)	0.338	9 (10.2)	14 (15.9)	0.371
male	111 (89.5)	74 (84.1)		79 (89.8)	74 (84.1)	
**Smoker, n(%)**						
no	36 (29.0)	30 (34.1)	0.527	25 (28.4)	30 (34.1)	0.515
yes	88 (71.0)	58 (65.9)		63 (71.6)	58 (65.9)	
**Brain metastasis, n(%)**						
no	104 (83.9)	72 (81.8)	0.836	71 (80.7)	72 (81.8)	1.000
yes	20 (16.1)	16 (18.2)		17 (19.3)	16 (18.2)	
**Lung metastasis, n(%)**						
no	115 (92.7)	85 (96.6)	0.372	82 (93.2)	85 (96.6)	0.494
yes	9 (7.3)	3 (3.4)		6 (6.8)	3 (3.4)	
**Bone metastasis, n(%)**						
no	102 (82.3)	66 (75.0)	0.266	74 (84.1)	66 (75.0)	0.191
yes	22 (17.7)	22 (25.0)		14 (15.9)	22 (25.0)	
**Liver metastasis, n(%)**						
no	89 (71.8)	79 (89.8)	0.003	79 (89.8)	79 (89.8)	1.000
yes	35 (28.2)	9 (10.2)		9 (10.2)	9 (10.2)	
**Type of ICIs, n(%)**						
anti-PDL1	86 (69.4)	53 (60.2)	0.218	61 (69.3)	53 (60.2)	0.269
anti-PD1	38 (30.6)	35 (39.8)		27 (30.7)	35 (39.8)	
**BED, Gy, Median(IQR)**						
NA	60.00 [51.75, 72.00]	60.00 [51.75, 72.00]	0.734	60.00 [53.10, 72.00]	60.00 [51.75, 72.00]	0.114

Abbreviation: PSM, propensity score matching; IQR, interquartile range; BED, biological effective dose; TRT, thoracic radiotherapy; ICIs, immune checkpoint inhibitors.

Supplementary Table 5. Baseline characteristics of subsets with BED≥60Gy and BED<60Gy in TRT group before and after PSM.

Characteristics	Before PSM			After PSM		
	BED≥60Gy(N=107)	BED<60Gy(N=96)	p	BED≥60Gy(N=75)	BED<60Gy(N=75)	p
**Age, n(%)**						
<65 years	69 (64.5)	59 (61.5)	0.764	49 (65.3)	49 (65.3)	1.000
≥65 years	38 (35.5)	37 (38.5)		26 (34.7)	26 (34.7)	
**Gender, n(%)**						
female	13 (12.1)	14 (14.6)	0.762	9 (12.0)	10 (13.3)	1.000
male	94 (87.9)	82 (85.4)		66 (88.0)	65 (86.7)	
**Smoker, n(%)**						
no	30 (28.0)	34 (35.4)	0.328	23 (30.7)	24 (32.0)	1.000
yes	77 (72.0)	62 (64.6)		52 (69.3)	51 (68.0)	
**Brain metastasis, n(%)**						
no	89 (83.2)	78 (81.2)	0.861	65 (86.7)	59 (78.7)	0.281
yes	18 (16.8)	18 (18.8)		10 (13.3)	16 (21.3)	
**Lung metastasis, n(%)**						
no	101 (94.4)	90 (93.8)	1.000	71 (94.7)	70 (93.3)	1.000
yes	6 (5.6)	6 (6.2)		4 (5.3)	5 (6.7)	
**Bone metastasis, n(%)**						
no	86 (80.4)	74 (77.1)	0.689	57 (76.0)	62 (82.7)	0.420
yes	21 (19.6)	22 (22.9)		18 (24.0)	13 (17.3)	
**Liver metastasis, n(%)**						
no	88 (82.2)	75 (78.1)	0.576	66 (88.0)	60 (80.0)	0.265
yes	19 (17.8)	21 (21.9)		9 (12.0)	15 (20.0)	
**Type of ICIs, n(%)**						
anti-PDL1	81 (75.7)	52 (54.2)	0.002	49 (65.3)	49 (65.3)	1.000
anti-PD1	26 (24.3)	44 (45.8)		26 (34.7)	26 (34.7)	
**Timing of TRT, n(%)**						
concurrent	44 (41.1)	42 (43.8)	0.813	26 (34.7)	29 (38.7)	0.735
consolidative	63 (58.9)	54 (56.2)		49 (65.3)	46 (61.3)	

Abbreviation: PSM, propensity score matching; BED, biological effective dose; TRT, thoracic radiotherapy; ICIs, immune checkpoint inhibitors.

Supplementary Table 6. Baseline characteristics of subsets with BED>39Gy and BED≤39Gy in TRT group before and after PSM.

Characteristics	Before PSM			After PSM		
	BED>39Gy (N=175)	BED⩽39Gy (N=28)	p	BED>39Gy (N=56)	BED⩽39Gy (N=28)	p
**Age, n(%)**						
<65 years	109 (62.3)	19 (67.9)	0.722	33 (58.9)	19 (67.9)	0.578
≥65 years	66 (37.7)	9 (32.1)		23 (41.1)	9 (32.1)	
**Gender, n(%)**						
female	25 (14.3)	2 (7.1)	0.463	10 (17.9)	2 (7.1)	0.321
male	150 (85.7)	26 (92.9)		46 (82.1)	26 (92.9)	
**Smoker, n(%)**						
no	54 (30.9)	10 (35.7)	0.768	21 (37.5)	10 (35.7)	1.000
yes	121 (69.1)	18 (64.3)		35 (62.5)	18 (64.3)	
**Brain metastasis, n(%)**						
no	143 (81.7)	24 (85.7)	0.804	46 (82.1)	24 (85.7)	0.918
yes	32 (18.3)	4 (14.3)		10 (17.9)	4 (14.3)	
**Lung metastasis, n(%)**						
no	165 (94.3)	26 (92.9)	1.000	53 (94.6)	26 (92.9)	1.000
yes	10 (5.7)	2 (7.1)		3 (5.4)	2 (7.1)	
**Bone metastasis, n(%)**						
no	142 (81.1)	18 (64.3)	0.075	36 (64.3)	18 (64.3)	1.000
yes	33 (18.9)	10 (35.7)		20 (35.7)	10 (35.7)	
**Liver metastasis, n(%)**						
no	142 (81.1)	21 (75.0)	0.615	42 (75.0)	21 (75.0)	1.000
yes	33 (18.9)	7 (25.0)		14 (25.0)	7 (25.0)	
**Type of ICIs, n(%)**						
anti-PDL1	115 (65.7)	18 (64.3)	1.000	31 (55.4)	18 (64.3)	0.584
anti-PD1	60 (34.3)	10 (35.7)		25 (44.6)	10 (35.7)	
**Timing of TRT, n(%)**						
concurrent	76 (43.4)	10 (35.7)	0.575	20 (35.7)	10 (35.7)	1.000
consolidative	99 (56.6)	18 (64.3)		36 (64.3)	18 (64.3)	

Abbreviation: PSM, propensity score matching; BED, biological effective dose; TRT, thoracic radiotherapy; ICIs, immune checkpoint inhibitors.

Supplementary Figure 1

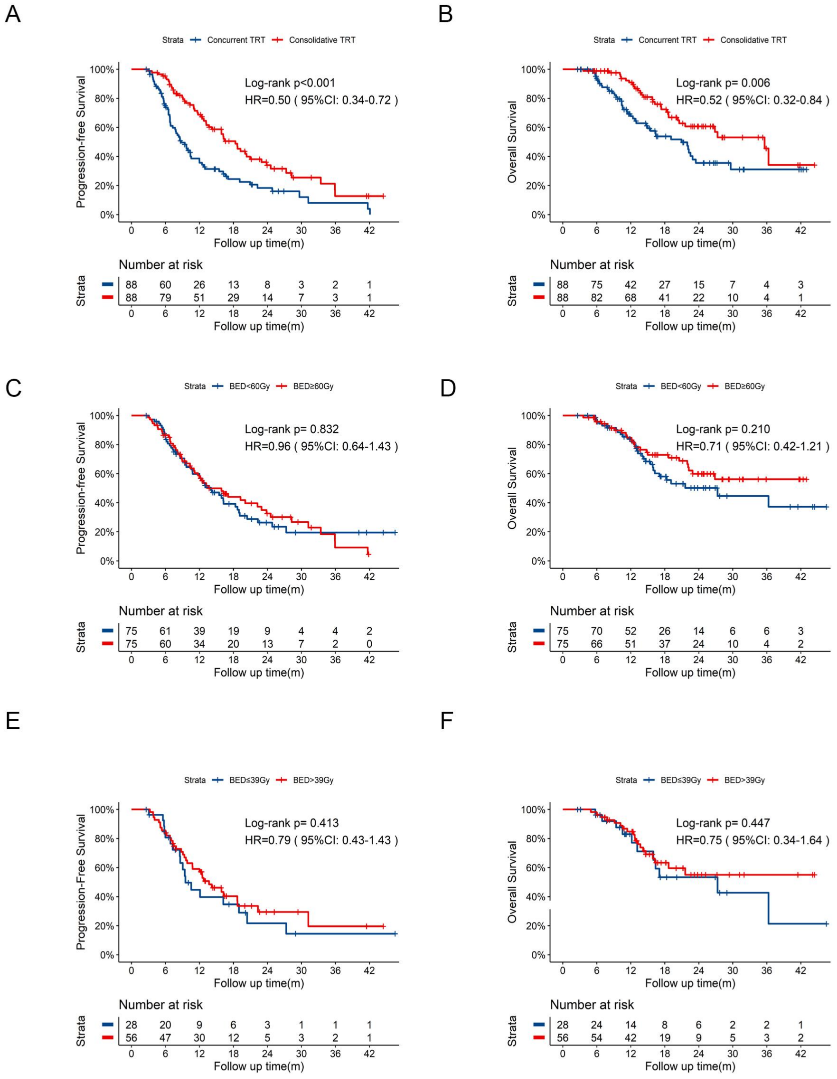

Supplementary Figure 1. Kaplan-Meier survival curves of different irradiation timing and doses in TRT group after PSM.

(A) Kaplan-Meier curve of PFS in concurrent TRT subset compared to consolidative TRT subset after PSM.
(B) Kaplan-Meier curve of OS in concurrent TRT subset compared to consolidative TRT subset after PSM.
(C) Kaplan-Meier curve of PFS in BED<60Gy subset compared to BED≥60Gy subset in TRT group after PSM.
(D) Kaplan-Meier curve of OS in BED<60Gy subset compared to BED≥60Gy subset in TRT group after PSM.
(E) Kaplan-Meier curve of PFS in BED≤39Gy subset compared to BED>39Gy subset in TRT group after PSM.
(F) Kaplan-Meier curve of OS in BED≤39Gy subset compared to BED>39Gy subset in TRT group after PSM.
PSM, propensity score matching; BED, biological effective dose; PFS, progression-free survival; OS, overall survival; HR, hazard ratio; CI, confidence interval; TRT, thoracic radiotherapy; m,months.
